# Dietetic Students’ Drivers and Barriers to Healthy Eating While Studying to Be a Healthcare Professional (a Pilot Study)

**DOI:** 10.3390/healthcare9050579

**Published:** 2021-05-13

**Authors:** Marie Trahearn, Dave Merryweather, Farzad Amirabdollahian

**Affiliations:** 1 The University Hospital of North Midlands NHS Trust, Stock on Trent ST4 6QG, UK; marie_021@hotmail.co.uk; 2 School of Social Sciences, Liverpool Hope University, Hope Park, Liverpool L19 9JD, UK; Merrywd@hope.ac.uk; 3 School of Health Sciences, Liverpool Hope University, Hope Park, Liverpool L16 9JD, UK

**Keywords:** dietetic students, barriers, drivers, healthy eating, food choice, qualitative

## Abstract

Background: For Dietetics students, starting university means developing the knowledge and skills required to be a healthcare practitioner. This pilot study aimed to explore the perceptions and views of the students on their drivers and barriers of healthy eating while studying Dietetics at university. Methods: A qualitative study was undertaken with a purposive sample of six final year Dietetic students at a UK university. Semi-structured in-depth interviews were used to elicit students’ experiences and perceptions of barriers to healthy eating. Interview data were analysed thematically. Results: Five themes emerged from the interview data including studying Dietetics, placement, influence of significant others, food security, and social and cultural aspects of the university life, with several sub-themes, and perspectives about the future beyond the university life. Conclusions: The findings suggest a potential need for Dietetics course providers to consider the range of barriers to healthy eating that students may encounter whilst studying and how these may undermine their ability to develop healthy eating practices and effective professional skills. Further research is required that explores the extent of barriers to healthy eating and examine whether these impinge upon effective practice.

## 1. Introduction

For many young people, starting university is a time of transition [1] when they begin to develop new skills such as cooking, shopping, and budgeting [2] and accrue greater control over their food choices [3,4]. For many, however, this can translate into unhealthy dietary practices [5], whereby missing meals [6], eating convenience foods and unhealthy snacks [7,8] and inadequate consumption of fresh fruit and vegetables [9] become the dietary norm.

Such poor dietary practices can be a consequence of various barriers that students as a whole may encounter. Greaney et al.’s study of American college students [10], for instance, identified lack of time, increased availability of unhealthy food on campus, peer influences and the expense of healthier foods as contributing to poorer dietary practices. Driskell et al. [11] also found college students to be more likely to eat in fast-food restaurants or university cafeteria than they were to prepare their own food; where convenience and cost again being cited as the key factors which limited healthy eating. These barriers are also encountered by students studying health-related courses. Nutrition students included in the Driskell et al. [11] study were found to be just as likely to eat unhealthily as other students, despite possessing good nutritional knowledge. Similarly, a study conducted in Pakistan found both medical and non-medical students to be equally likely to consume fast-food and eat unhealthily, both cohorts citing a lack of time and the stresses of academic study as key factors restricting their diet [12]. Research into Dietetics students at a Canadian university likewise concluded that a lack of time to prepare healthy meals and a paucity of food choice on campus severely limited their ability to eat healthily [13].

Barriers to healthy eating and lifestyle encountered at university will inevitably have health-related consequences for students in general but there might be further implications for those studying to become Health Care Professionals (HCPs) [12]. As such, it is important to understand the pressures faced by these students as well as the implications of this for their professional practice. However, little is currently known of the dietary practices of health and medical students studying in the UK.

We have been fascinated by the complexity of making food choice amongst the Dietetics students, and in order to produce a conceptual framework for our work, we explored the phenomenon within a model of food choice, and also more broadly as part of a theoretical model explaining the health behaviours of the individuals in general:

The individuals’ food choice is a result of a complex set of processes and influencing factors [14], working simultaneously and sometimes subconsciously to regulate eating behaviour [15]. In an attempt to understand the processes involved; many models have been developed to explain the key factors that can influence an individual’s food choice [16]. However, there is no globally accepted model that can capture all aspects of the complexity which surrounds food choice [17].

**Figure 1:**
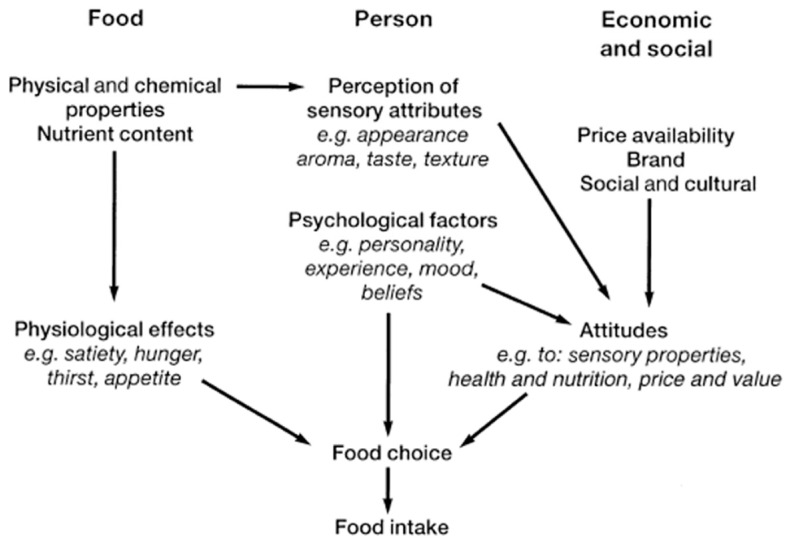
Shepherd (1985) model summarizing factors affecting food choice and intake adopted from Shepherd (1999) [19].

An early research by Yudkin (1956) proposed that factors influencing food choice could be divided into three main categories; physical (e.g., geography and season), social (e.g., religion and social class) and physiological factors (e.g., therapeutic diets and nutritional needs) [18]. However, a later food choice model developed by Shepherd (1985) included more emphasis on the role of ‘individual’ factors such as personality and experience in food choice (Figure 1), postulating that the influencing factors can be divided into three main categories relevant to the food itself, the individual making the choice, and their economic and social environment [19]. The subsequent publications [20,21,22,23,24] produced further definitions and clarifications for the elements of the Shepherd’s model, explaining that the ‘food itself’ in this model is concerned with the nutritional content and also physical and chemical properties of the food, and the ‘personal’ factors are concerned with how food is perceived to the individual; for example, its taste and texture. On the other hand, the psychological factors are very individual to each person as this comprises the individuals’ attitude, mood and levels of stress, and these psychological factors can influence an individual’s emotions and feelings toward a certain food. Finally, the socioeconomic factors in this model are concerned with the environment and social surroundings of the individual, including the availability, accessibility, and affordability elements of the food security. Within this model, the psychological, the personal and the socioeconomic factors all interrelate to shape an individuals’ attitude toward food.

In this study, we used Shepherd (1985) as the core conceptual model for exploring the food choice of the Dietetic students [19]. The model was selected as a good fit for our qualitative research considering its strength that rather than merely stating the factors which influence food choice, it attempts to explain how they are related. For instance, it explains how an individual can develop a learned association between the nutritional content of the food (e.g., the amount of protein it contains), and the physiological consequences that the food causes (e.g., how well it reduces their hunger); through experience, this process allows food preferences to develop, which in turn will affect an individual’s food choice [19]. Another advantage of this model in comparison with other models such as Yudkin (1956) [18] is the fact that the model considers the actual ‘food intake’ as a result of the food choice. We felt that this is important as once the food has been chosen; further decisions are to be made such as the portion size of the food to be consumed and many factors can affect this decision [25].

**Figure 2:**
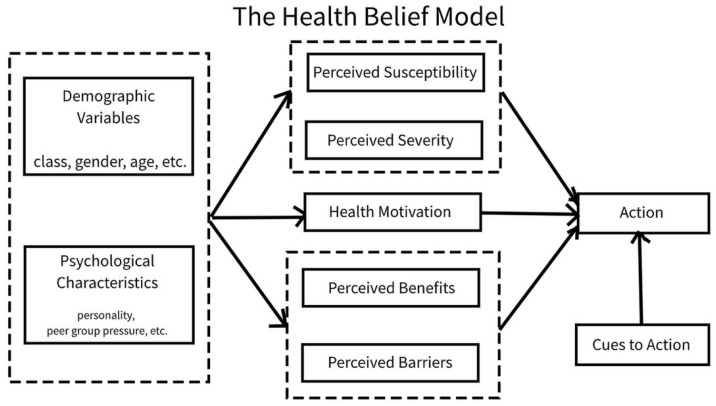
The Health Belief Model (HBM) by Rosenstock (1966) [26] was used in this study to explore the factors driving and inhibiting making healthy food choice. The diagram is produced by an unknown author and licensed under CC BY-SA for public use.

While we implement the model of Shepherd to explore food choice, to further explore the factors enabling or inhibiting making ‘healthy’ food choice within our study, we used the Health Belief Model (HBM). The HBM (Figure 2), proposed by Rosenstock (1966) [26] is a widely used and established model explaining the health behaviour. The perception of threat and the evaluation of the behaviour are the key components of the HBM [27]. The threat perception is postulated to include perceived susceptibility and perceived severity, the first in relation to the belief about the likelihood of being affected by the negative consequences of the behaviour, and the second in relation to the belief about the likelihood of the seriousness of those negative consequences [27]. To conceptualize the current study within the context of the HBM, the Dietetic students threat perception could include their beliefs about how vulnerable they are to the adverse effects of poor eating behaviour (e.g., ‘my diet could put me at risk of diabetes in later life’) and how severe the impact of this is likely to be (e.g., ‘diabetes could lead to serious consequences and kill me’), and conceptually if the overall perception of the threat, can lead to the belief that their health may be endangered, the likelihood of changing behaviour and improving toward a more healthy eating can be increased. To further examine the behaviour evaluation component, the model refers to the balance between the individual’s perceived benefits and barriers of the change of the health behaviour. Therefore, if our Dietetic students believe that healthy eating will improve body shape, wellbeing, academic performance, etc., these collective beliefs about the benefits will increase the likelihood of improving and adherence to healthy eating behaviour. In contrast, Dietetic students are also likely to hold some beliefs about potential barriers to change toward healthy eating (e.g., ‘My family may not support my newly developed eating habits’), and if they feel that they can overcome these barriers (e.g., I will talk to my family to explain what I know about this diet) or if in the individual’s ‘weighing up’ of perceived benefits compared with barriers, the benefits outweigh the barriers, then the behaviour evaluation process of the eating behaviour can lean in favour of healthy eating behaviour [27]. While we have briefly outlined the two main components of the HBM, it is also imperative to outline other influences within the HBM as they also substantially contribute to the determination of the behaviour. For instance, the HBM proposes that the health behaviour is influenced by the individual’s health motivation and suggests that the ‘cues for action’ can impact health behaviour. For our Dietetic students, we postulated that combined external and internal cues for action frequently received from the living, learning and working environment especially while experiencing similar/familiar background, experiences and symptoms to patients (e.g., ‘I am also always exhausted, and this is because I am overweight, the same as the patient who was complaining about this in the ward today’) can be key determinants of the healthy eating behaviour [27].

This small-scale pilot study aimed to provide an understanding of the perceptions and views of the Dietetic students on their drivers and barriers of healthy eating while studying Dietetics at university. Specifically, researchers were concerned with how aspects of student life, academic study and work placement impacted upon students’ eating habits. As a course which trains students to become National Health Service (NHS) professionals who have a responsibility for promoting healthy eating and improving the population’s health [28], we speculated that Dietetics students would have a strong desire to follow a healthy diet and lifestyle and indeed promote it. We assumed that preliminary investigation identifying factors affecting the eating habits of Dietetics students can lead to generation of surveys and questionnaire to further explore this within the broader Dietetic student populations and enable health education providers to improve training with a view to helping practitioners and service users improve their eating habits.

## 2. Materials and Methods

### 2.1. Design

The present pilot study utilised a qualitative approach to develop a preliminary understanding of key barriers to healthy eating by using methods that privilege the experiences, perceptions, and assumptions of research participants [29,30,31]. While such an approach has been demonstrated to be useful in examining food behaviours, drivers, and barriers to healthy eating amongst the general population [32,33], it has only recently been applied to the field of Dietetics [34,35]. With this background, the qualitative approach was used as conceptually the study intended to generate thick, rich, and descriptive narrative data to gain a deeper understanding of the individuals’ thoughts, feelings, beliefs, and attitudes towards a specific phenomenon [31,36]. A process of inductive reasoning was, therefore, commonly used to build theory where there is a lack of existing knowledge in the subject area [37]. From design perspectives, this was an observational study which adopted a phenomenological approach. Other approaches were also considered by the researchers, such as ethnography [38]; however, a phenomenological approach was deemed most appropriate due to being more concerned with exploring an individual’s lived experiences of barriers and enablers of healthy eating through their own words [39].

### 2.2. Recruitment

Dietetics students were identified as potential research participants using purposive sampling. These students were known to have knowledge of the phenomenon being investigated [40] and, furthermore, to be able to generate appropriate data based on their specific experiences [41]. They were also required to be able to read and speak English to understand the participant information sheet and provide written consent for participation in the study. Other than being Dietetics students and adherence to the ethical approval, the only other inclusion criterion was that they should be in their final year of study. This was to ensure a degree of parity as regards knowledge and experiences of healthy eating and dietary practices acquired over the course of their study. For the purposes of recruitment, a member of the research team attended a Dietetics lecture, informing students of the study’s aim, and requesting volunteers to participate. Using this approach, six students out of the cohort of thirty-four indicated a willingness to take part.

### 2.3. Data Generation and Instruments

Semi-structured in-depth interviews were conducted for the purposes of generating data. This method was favoured as it allowed a more informal, flexible approach to exploring participants’ thoughts and feelings of the research topic [31] and enabled researchers to develop an understanding of drivers and barriers to healthy eating based upon student Dietitians’ experiences, perceptions and constructions of their own social realities [29]. Each interview took place in a small interview room within the university. This provided a convenient and familiar research environment which minimised the risk of interruptions [40] and facilitated in-depth discussions.

**Figure 3:**
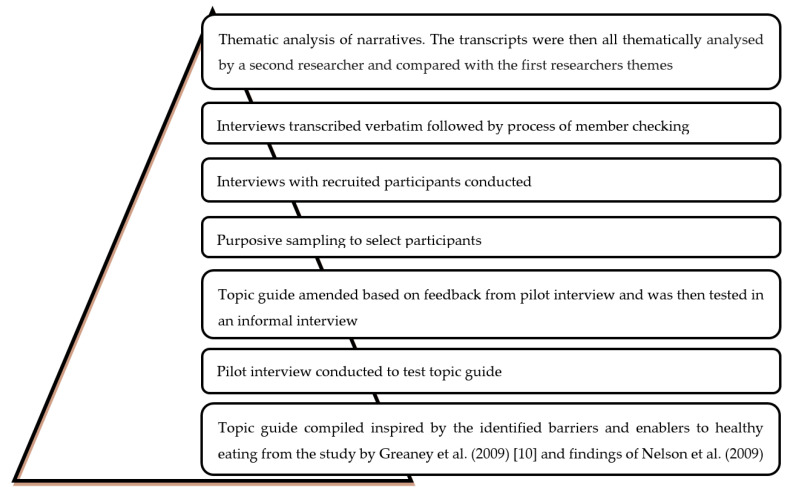
Process undertaken for establishing the topic guide for the semi-structured in-depth interviews.

Interviews were conducted using an interview guide [42], a general framework which, rather than detailing a prescribed list of specific questions, outlined the core topics to be covered. This ensured that areas of interest to participants were included and allowed the researcher to prompt and probe so, as to elicit more in-depth responses where necessary. A summary of steps taken to produce, pilot and finalise the topic guide can be seen in Figure 3 and the final topic guide can be seen in Table 1. Interviews were recorded on a Dictaphone (Olympus DS-550, OM Digital Solutions GmbH, Hamburg, Germany) so as to ensure that all data were captured, thereby facilitating subsequent transcription and analysis [43].

### 2.4. Data Analysis

All interviews were transcribed verbatim and subjected to thematic analysis [44,45]. This entailed repeated detailed readings of the transcripts to identify key patterns of experience and understanding of dietary practices, food choices and barriers to healthy eating. These were grouped together into specific themes [42], which were then cross-checked by other members of the research team to ensure validity of the data. Transcripts were then compared so, as to elucidate the ways in which the key themes resonated across the data.

### 2.5. Ethical Approval

Ethical approval was granted by Coventry University’s Faculty of Health and Life Sciences Ethics Committee. All participants were required to read a participant information sheet providing detailed information about the research and to sign a consent form.

## 3. Results

Final year Dietetic students were included in the current study, including five female students and one male student. A summary of the participants characteristics can be found in Table 2.

The key themes and sub-themes are shown below along with quotations which have been taken directly from the interview transcriptions. To illustrate these themes, the narratives were coded to enable the quote to be located within the transcriptions. The alphabet and digit in brackets have been used to relate the quote to the participant and line number of the transcripts. Table 3 provides additional quotations from participants to further demonstrate the themes. Dietetics students identified numerous factors influencing their eating behaviours and attitudes in relation to diet and food choices. This included studying Dietetics, placements, influence of significant others, food security, and social and cultural aspects of university life, while all participants further discussed their perspectives about their eating behaviour beyond the study of the Dietetics.

### 3.1. Studying Dietetics

#### 3.1.1. Weight

When Participants B, C and F started the Dietetics course, they felt they needed to lose weight to conform to the image of a dietitian, which resulted in increased exercise and dieting behaviours. Participant B began recording exercise and dietary intakes in a logbook in an attempt to lose weight.

*‘I was eating like literally 600 calories a day and like the course gave me the knowledge...so I had a better understanding of how to calorie count.’*

(C92)

#### 3.1.2. Knowledge and Awareness

All participants felt they had more knowledge due to studying Dietetics and were more aware of the evidence and recommendations to be able to lead a healthy diet. As a result, participants C, E and F made positive changes to their diet such as more regular meals and eating more fruit and oily fish.

*‘...when we did about the importance of omega 3 in the body...it triggered me to try and eat more oily fish and take omega 3 supplements’*

(E54)

Studying a Dietetic course and learning about nutrition and lifespan prompted Participant D to start her children on school meals due to the benefits of social eating for children; this in turn resulted in evening meals being missed due to not being required to cook a meal for her family (D32).

It is important to consider that participants typically spoke of having held positive attitudes towards diet and as engaging in healthy eating practices prior to commencing study of Dietetics. This was often characterised by the eating of healthy meals prepared by parents, and by structured mealtimes.

*‘Obviously my mom cooked all my meals and because I went to sixth form as well, I’d have breakfast before I went to school and then I’d either buy lunch at sixth form or I’d take a packed lunch with me sometimes and then have a snack when I got home. And then there were like set meal times so it was quite structured and I didn’t really snack very often because I knew that my meal would be later so I wouldn’t be hungry for it.’*

(B16)

Prior knowledge of healthy diet was typically augmented by the course itself, several noting that they had been endowed with additional information and skills that had enabled them to adopt a healthier diet and to make more positive food choices.

*‘[the course] makes you more aware of what you’re doing to your body and makes you want to try and be more healthy and obviously makes you more aware of the content of each food such as the calories, and the fat and the sugar content so that can sometimes sway which way for the foods I’ll pick. Like, for example, generally I like to go for the more healthier options like a sandwich but the course has made me like look at the labels more and yeah just be more aware really…*

(A98)

#### 3.1.3. Time

Participants A, C, D and F reported a lack of time to make healthy meals due to the workload of the course which sometimes causes them to choose convenience foods. A lack of time for Participant D means she chooses quick meals and as a result consumes a more vegetarian diet.

*‘...it was more healthy before because I had the time to prepare it so I was including more meat’*

(D52)

Timetabling of lectures was cited as a major contributor to the consumption of unhealthy snacks or convenience foods as well as the skipping of meals.

*‘Lectures like are at different time every day… [and so] I might have a really late dinner or a really late lunch or I might skip lunch or eat out… It’s really unstructured.’*

(B34)

#### 3.1.4. Stress

All Participants saw various aspects of the study and university life as having at least some negative impact upon diet and food choices. Participants regarded the various demands of the course itself as having detrimental consequences for dietary practices. Participants B, C, D, E and F described how they choose convenience foods and unhealthy snacks when they are stressed due to the workload of the course. Participant D gave up smoking whilst at university which caused her to select more unhealthy snacks when she is stressed but along with Participant F also chooses healthy snacks to try and compensate.

*‘Like the amount of stress itself has gone up at university with like the work and things and trying to fit everything in and trying to balance everything out, so yeah being stressed definitely makes me eat more and it’s mostly chocolate and sweets and things like that and it wouldn’t really be very healthy to be honest it always makes me eat more unhealthy.’*

(E126)

Participants D and F frequently become despondent which causes them to comfort eat; for participant F, this has turned into a daily binge on snacks at university, which has resulted in weight gain:

*‘It’s due to practically binging on food daily, a gain of 7 and a half stone so it’s a lot.’*

(F76)

### 3.2. Placements

All participants distinctly referred to the placement as an important factor contributing to food choice and eating behaviour.

#### 3.2.1. Food Shopping While at Placement

Participant C; who moved accommodation on placement, reported that particularly during the placement period without a car, food shopping was difficult and this was similar for Participant F as it limited where she could do her food shopping:

*‘The supermarket was miles away literally like we were in the middle of no-where, we didn’t shop very often... so I used to eat the same things every day.’*

(C180)

#### 3.2.2. Meal Pattern/Structure While at Placement

Participant E found a lack of planning meals on placement resulted in unwanted weight loss (E134) while the amount of travelling also affected his food choices.

*‘...it’s hard to eat when you’re on buses and trains and stuff as well...you can hardly have any variety as well, like sometimes I’d take my dinner with me and it would be like cold pasta with tuna and stuff whereas at home it would be a hot meal.’*

(E138)

Not all participants considered their Dietetic placements in such negative terms and for some, this afforded a more structured routine that enabled the planning of meals and regular meal times. This was augmented for those living in nursing accommodation in or in close proximity to the placement location where affordable and healthier food options were more readily available. Due to absence of their peers as well as having less control over their food choices, participants A and B found placement provided a more structured meal pattern with less snacks due to meals being cooked for them and the working hours providing set mealtimes:

*‘All my meals were cooked for me... this really changed my diet because I just had three set meals provided... so I wasn’t eating any junk.’*

(B220)

Nonetheless, not all students were so fortunate and clearly the type and location of placement had a potential to encumber dietary practices commensurate with the ideal of being a dietitian.

### 3.3. Influence of Significant Others

#### 3.3.1. Peers

The influence of friends and fellow students was also regarded as a factor affecting dietary habits. For some, this influence was positive in that eating and cooking habits had improved.

*‘I’ve been exposed to different people’s diets and the foods they have like curries and hummus and lots of different things, so I feel that I have a wider variety of food now.’*

(B64)

Participants B and F would eat more healthily around their peers due to the fear of being judged as a Dietetic student. However, for the most part, students saw this influence as being a negative one.

*‘I live with dietitians [laugh] so I feel they could be quite judgmental sometimes if I was eating loads of junk food. That would be another reason why I might try to eat healthily around them.’*

(B60)

Others considered that there was a greater likelihood of consuming fast-food, unhealthy snacks, desserts, and alcohol when in the company of friends who made unhealthy food choices:

*‘I’d say they’re [friends] a bad influence (laugh) um just because they sort of have cravings for bad food at times where maybe I’m eating healthily but in the end sort of I’ll give in and I’ll end up eating unhealthily options when they do, so more often. Like now I’d say I have take-a-ways about two or three times a week on average when before, like when I was at home, it was probably more like three or four times a month (laugh) …’*

(A76)

Participant C reported being pressured to eat larger portions than she would normally have.

*Her [flatmate’s] diet isn’t very healthy (laugh) and sometimes she influences what I eat in terms of portion sizes as well. Because she eats a lot she sometimes forces me like ‘is that all you’re eating?’ I feel pressured and if like she’s having say a biscuit, I’ll be like go on then, it’s like the peer influences which I think isn’t very good…’*

(C74)

#### 3.3.2. Family

Participants A, B, C and F reported less parental control at university resulted in a more unstructured meal pattern and an increased consumption of unhealthy snacks:

*‘My mom was quite strict like she would lock all the chocolate away...now it’s like ‘because I can have it I have loads of it’*

(B46)

However, Participant E felt his partner had a stronger influence over his food choices due to exposing him to different types of foods.

### 3.4. Food Security

#### 3.4.1. Accessibility

Participants A, C, D and E commented on the advantage of having access to a car to enable greater accessibility to the supermarket which gave them a healthier diet.

*‘I had a car...that made it more accessible for me to go and get bulk storage items and... fresh stuff which obviously weighs quite a lot.’*

(A72)

Without a car, participants B, C, E and F relied upon the local shops, markets, and small supermarkets. Participants B and C commented on how the easy access to local shops resulted in them not having to plan meals in advance and allowed them to buy unhealthy snacks at any time. Participant E reported that due to the difficulty carrying the food home, she was only able to purchase the items which did not weigh a lot. Participant D had easy access to the local shops due to a car and also now relies on them more now for her food shopping rather than the larger supermarkets (D86), partly due to the amount of food that was wasted from a large shop and due to a limited budget at university.

#### 3.4.2. Affordability

Participants B and E reported the smaller shops are expensive and lack a variety of healthy options. Participants C and F felt that it would be beneficial for healthy food to be more available on campus and in the small shops:

*‘…maybe partnerships with places...to provide cheaper produce for students.’*

(C194)

Due to the distance and difficulty carrying food home, Participant F used online shopping to allow a greater amount of food to be purchased.

#### 3.4.3. Availability of Storage Space

A lack of storage space for food in university accommodation resulted in some participants having to think carefully before purchasing large items as, for example, Participant A had to compete for fridge and freezer space.

*‘it was appalling really we just had a tiny fridge, so it was really hard to store fresh fruit and vegetables... it sort of prevents you shopping on a monthly or weekly basis, you shop more day to day’*

(B112)

The lack of storage space left the participants limited with the amount of food they could purchase; this caused participants A, B, E and F to go food shopping more regularly and caused Participant A to consume more fast-food. Participants B, E and F reported purchasing more dried and tinned foods rather than fresh due to the lack of space.

### 3.5. Social, Lifestyle and Cultural Aspects of University Life

Food choices were influenced by other social and cultural aspects of university life. Participants reported a newly found freedom of health behaviour within the university lifestyle, and also on how the university drinking culture affected their dietary habits, acknowledging variously that they consumed less food before going out drinking, ate more fast-food following alcohol consumption, or skipped meals the following day on account of having a hangover or to compensate for the calorie intake of the drinking night out. For most participants, however, the frequency of drinking and associated poor food choices had diminished as they had progressed into the final year of study, particularly in light of having learnt more about the various health risks associated with excessive alcohol consumption and unhealthy food choice.

#### 3.5.1. Freedom to Make Food Choice

For some participants, simply moving into university accommodation and living independently from parents had led to less structured and often unhealthier dietary practices:

*‘There’s no-one telling me what to eat anymore, it’s much more my choice so if I want something then I can have it. I suppose ‘cause at home my mom was quite strict like she would lock all the chocolate away and things [laugh], now it’s like ‘cause I can have it I have loads of it [laugh] so I eat a lot more unhealthily.’*

(B46)

*‘At home I was not allowed sugary breakfast cereals at all, but at university the first thing I bought was sugary cocoa pops to have for breakfast.’*

(B104)

#### 3.5.2. Eating out, Fast Foods, and on-Campus Food Establishments

The newly found freedoms and practical restrictions often translated into dietary practices which centered on fast-food outlets and all-you-can-eat restaurants; establishments which participants recognised as offering less than healthy food choices. For instance, participants’ A, B, C, E and F mainly used the cafes and food establishments on campus in their first years due to them being convenient.

*‘They don’t actually have much variety, most of them [sandwiches] have all got mayonnaise in so I think they do need more um healthy options.’*

(A90)

*‘I think they are quite expensive and I’ve noticed that they are erm lacking in healthy options… there is no fruit and vegetables at all in there really.’*

(F80)

Participants also reported routinely eating out or consuming more takeaways and fast food at university as part of the university culture and lifestyle:

*‘I’d get like chips and then either like burgers and battered sausage...and things like that after we’ve been out.’*

(E106)

*‘When I was in the halls in fact there were a lot of take–a-ways literally directly across the road so if a lot of my friends in the halls would be getting food then we would obviously all get it together so whereas I would have made a healthy meal for myself I kinda joined and went for the more unhealthy option’.*

(A68)

Despite this, participants claimed to use fast food outlets far less frequently in their final year of study, largely on account of an increased recognition of the poor quality of such food, high costs and lack of variety of healthy options.

#### 3.5.3. Alcohol

Compared to before university, all participants reported consuming more alcohol and frequently binge drinking in their first year due to the influence of their peers and the alcoholic drinks being cheap. This affected their dietary habits, causing them to either consume less food before a night out, eat more fast-food following alcohol consumption or not eating anything the following day due to a hangover. Participants felt their drinking habits have improved throughout university due to realising the health risks associated with binge drinking (A38), apart from participant D.

*‘I’ll skip breakfast and I’ll get to possibly two three o clock and have something to eat then ... go all the way through and then just go out drinking.’*

(D66)

#### 3.5.4. Religion and Culture

Participant C fasted once a week and had to frequently change social plans to be able to continue with this at university (C140).

*‘It’s been hard, especially if you want to go on a night out or something. If someone is planning a meal or something I’d always be like [pause] because I have to be a vegetarian on that day I’m not allowed to eat meat so I’d be like ah ‘can you do it on another day’*

(C140)

#### 3.5.5. Physical Activity

Participant E began training for sport at university, which had the biggest influence over his diet; relying on snacks like chocolate to help him gain weight. Participant B also distinctly referred to running and adjustment of eating habits to suits this new health behaviour.

*‘I think I probably eat more for like sport reasons to gain weight, because I was really skinny before erm, so I’ve been trying to eat more and go to the gym, that’s been really important to me that’s been my main influence’*

(E42)

### 3.6. The Future Beyond University

#### Increased Awareness of Barriers to a Healthy Diet

All participants commented on how they have become more appreciative of other people’s dietary habits and difficulties people can face when trying to lead a healthy diet. All participants felt they would be able to use their experience of how university had impacted on their diet positively to think of innovative ways and give advice to their patients to help them overcome barriers to leading healthy diets (D152).

*‘I think it’s made me understand a bit more especially if I was seeing young people who were students like the difficulties and barriers that I’ve had myself’*

(E153)

## 4. Discussion

The results of this small-scale pilot study suggest that knowledge and awareness, together with psychological, and socio-economic factors, are the main determinants of Dietetics students’ dietary habits. Interpreting these findings within the framework of the Shepherd’s model of food choice [19], while there are some clear narratives demonstrating the impact of the area of food and its physiological effects (mostly within the theme of studying Dietetics), the majority of findings relate to the personal and socioeconomic parts of the model. The strong narratives about the role of food security (from accessibility, availability, and affordability perspectives) and psychosocial factors (from perspectives about the role of significant others and compliance with the body image and ideals of being a Dietitian) together with narratives within the themes of social, lifestyle and cultural aspects of the university life highlight the importance of personal, social and economic factors in shaping the attitude of the Dietetic students toward food.

Students within our study possessed good understanding of healthy eating, often founded in previously acquired knowledge and experiences prior to the university life, and augmented through studying Dietetics. This was reflected in a strong desire to develop healthy dietary practices which corresponds to the ‘perceived benefits’ elements of the HBM [26]. Nonetheless, and again in line with the HBM, our findings suggest that participants encountered several barriers limiting the incorporation of this knowledge and understanding into their health-promoting practice.

A major barrier related to the impact of stress and lack of time associated with the pressures of university life. This echoes the findings of previous research identifying a strong link between stress and comfort consumption of sweets, chocolate, and convenient and fast foods [46] and pointing to the impact of time restraints upon preparation of meals and healthy eating [12,13,47]. The current study contributes to this by using qualitative data to demonstrate the depth of the impact of these barriers on healthy eating and their implications for the health and wellbeing of the students concerned.

A further finding was that friends and fellow students affected the types and the amounts of food consumed. The impact of such peer influence, also reported by Nelson et al. [5], corresponds to both matching norm and the norm for minimal eating suggested by Roth et al. [48] to explain the social influences on eating. Analysis of findings shows that Dietetics students frequently matched or modelled the food intake of peers with whom they were eating. Furthermore, the norm for minimal eating also appeared to exist (especially among female participants), implying that making a positive impression was important within this particular group.

The most significant finding relating to food security was that economic and socio-cultural influences around food availability also exercised an important influence over food choices. Dietary practices were often constrained by lack of adequate cooking and storage facilities, especially for those students living away from home or in accommodation linked to their placement. Additionally, not being able to use a car, limited access to larger supermarkets and over-reliance on more expensive local shops often meant participants had limited access to affordable healthy food options. This is consistent with research highlighting the importance students attribute to value for money, cost, and convenience when shopping for food [48].

There are a few strengths for this study. First of all, with regard to originality and contribution to the body of knowledge, while there are several previous studies examining nutrition (e.g., [8,49,50,51,52,53,54]) and health (e.g., [55,56,57,58,59,60]) of young adults, the majority of these studies use a quantitative approach to describe or quantify the issue, and a few well-conducted qualitative studies (e.g., [33,61,62,63]) are not focused on eating habits of the Dietetic students, as the future health care professionals. Our study explores the ‘how’ and ‘why’ questions of the eating behaviour of the Dietetic students using a qualitative approach to address the gap in our understanding in the food choice of the Dietetic students. Furthermore, our study interlinks with the theoretical perspectives and models of food choice and planned health behaviour to explore the barriers and drivers of healthy eating amongst Dietetic students as a specific population group. The rigor of the research conduct is the second strength of the current study as several steps were taken to enhance the rigor in line with the literature’s recommendations [32,64]. These included completing the interviews until data saturation and taking field notes comprising the researcher’s observations of the participants during the interviews to make sense of the information when it was being analysed. Additionally, member checking was conducted, and analyst triangulation was used as the transcripts were all thematically analysed by a second researcher and compared with the first researcher’s themes. The topic guide of the interview was also tested in a pilot interview to allow the opportunity for modifications.

There were, however, some limitations to this pilot study. Most importantly, only a small number of participants were interviewed and, consequently, it would obviously not be possible to generalise the results to the wider Dietetics student population. While this is the case, it might be worthy to remember that conceptually, unlike quantitative research, qualitative studies are not methodologically concerned with producing results which can be ‘generalised’ to a wider population as they are more concerned with providing information which could be useful to apply in other situations [64] and, hence, we have been more focused on qualitative transferability of the research in comparison with quantitative generalizability to wider populations.

The sample size of qualitative research has been an area of extensive debate and controversies. The extensively cited Drowkin (2012) work critically appraising the sample size policy for establishing qualitative in-depth interviews considered a large body of knowledge including articles, books, book chapters and qualitative guidelines who recommended the sample size anywhere between 5 and 50, but highlight to the importance of practical and methodological considerations in decision making about the number [65]. The systematic analysis of qualitative health research over a 15-year period to characterise and justify sample size sufficiency in interview-based studies conducted by Vasileiou et al. (2018) highlighted the extensive academic debate on the matter and demonstrated that the provision of sample size justification in qualitative research within the discipline to be limited, dependent on publisher’s guidelines and practices and often in view of the principles of saturations of information and pragmatic considerations [66]. We refer to the recommendations of Vasileiou et al. (2018) to first of all be transparent about the evaluation of the sample size sufficiency. As a small-scale pilot study, we recruited the six out of the thirty-four students available within the cohort mostly in view of financial constraints and pragmatic considerations. Considering the extensive workload of the final year Dietetic students and their limited availability, lack of access to transcription software packages (each interview taking up to eight hours to transcribe), short window of opportunity for completing the project and long duration of each interview for reaching the data saturation, and the pilot nature of the current work, we used the pragmatic considerations as our other counterpart researcher for deciding about our sample size. Despite this, through conducting and analysing the interviews, we felt that there was/is a large degree of saturation and information redundancy which gave us more confidence that the data can be adequate for the pilot as recommended by the literature [66]. While we categorically acknowledge our sample size to be a key limitation of our study, we reflected on a. the data saturation and the appearance of the information redundancy, b. the fact that we meet the minimum sample size number reported by Dworkin [65] (n = 5), and Vasileiou et al. [66] for comparable research from the British Journal of Health Psychology (n = 6), c. the richness and quality of the data obtained, and e. the fact that our research could inspire more extensive qualitative research and quantitative descriptive surveys deciding to disseminate the methodology and findings of our pilot in full transparency.

As the current study was conducted as a pilot to inform future qualitative studies and surveys, it is important to consider the time consuming and labour-intensive methods implemented, which required lengthy interviews, and extensive manual data transcription, member checking, triangulation, and thematic analysis. The future studies with larger samples size will require implementation of technological solutions that was not available to us. For instance, this can include the use of qualitative data analysis software packages (e.g., NVivo-QSR International Pty Limited, Melbourne, Australia) as part of a mixed methodological approach to facilitate the transcription and analysis.

The researchers have been conscientious about the potential influence of their own beliefs, experience, opinion and understanding on the views of the participants and the interpretation of the data, considering that two of the authors have been established university academics working with healthcare students for more than a decade, and a dietitian who used to be a Dietetic student, completed the fieldwork of the data collection. The process of reflexivity was used as a process for consideration of our researchers’ own role in the research process via critical self-scrutiny in line with the literature [32,64,67]. For instance, the interviewer took note of her own perspectives and assumptions prior to the interviews beginning and was particularly careful in her questioning by asking open and consistent questions so as not to lead the participants.

This is important to consider the findings of the current research with care and caution. For instance, the researchers recognise that to become a practicing dietitian, one must complete extensive training at university, as well as passing a number of professional assessments and clinical placements. Therefore, we do not claim that the barriers to healthy eating whilst at university would adversely affect their clinical practice as qualified dietitians but feel it would be beneficial for further research to be conducted to examine if there are any longer lasting effects of unhealthy dietary choices at university which may be impacting upon the Dietetic students in their later adult life.

## 5. Conclusions

This small-scale pilot study was undertaken to explore Dietetics students’ drivers and barriers to healthy eating and, to begin to develop an understanding of factors affecting Dietetic students’ food choice while studying at university. Participants encountered a wide range of barriers; course-related factors, accommodation type, storage facilities, cost, and availability of healthy food, all had a detrimental impact upon students’ capacity to fulfil the ideal of being a dietitian. These findings indicate clearly that further extensive research into this important issue is required. This should focus on developing improved knowledge and understanding of the full extent of such barriers. Furthermore, knowledge is required as to the impact of such barriers upon dietitians’ professional practice once they qualify as Health Care Professionals. Finally, the findings indicate a potential need for Higher Education Institutions to consider the social, economic, and psychological barriers and facilitators of healthy eating when planning and implementing strategies relevant to the training of future Health Care Professionals.

## Figures and Tables

**Table 1:** Topic guide used for interviews after completion of steps summarised in Figure 3.

Areas of Exploration	Prompts
Participant demographics	Questions about Age, Sex, Marital status, Number of children, Dietary restrictions/taking any supplements
Dietary habits before beginning university	Prompts about where participant lived and who did s/he live with, what participant’s diet was like before s/he started studying, the main influencing factors on his/her diet, and control over food shopping/preparation/cooking
Dietary habits at university	Prompts about describing the current diet and its balance, the difference to before university, the main influences on diet, and feeling towards diet at university
Accommodation, lifestyle, and place of residence	Prompts about if participant moved accommodation whilst at university, influence of accommodation on diet, number of people participants lived with, weight change at university and reason, change of shopping habits and reason, access to food while at university, the use of the on-site facilities, e.g., cafes, student unions, and the cooking/meal preparation habits
Dietary habits and potential influences	Prompts about any change of dietary habits during time at university If necessary, prompts about portion sizes/types of food, weekend dietary habits, habits continued since before beginning university, eating out and alcohol consumption. If following factors have not already been discussed; prompt as necessary on the key influences such as culture, religion, partners, family, mood, etc.
Dietetics course and its placements	Prompts about the knowledge and its impact on eating, the impact of timetable, accommodation, travelling, and working hours and their potential impact on eating
The future	Prompts about life and diet after university, what the participant foresee to change after university, things that the university could have done to support students, and the university eating experience and its impact on future career as a dietitian

**Table 2:** Characteristics of the participants of the study.

	Participant A	Participant B	Participant C	Participant D	Participant E	Participant F
Age (years)	23	22	23	34	22	23
Gender	Female	Female	Female	Female	Male	Female
Ethnicity	Caucasian	Caucasian	Asian	Caucasian	Caucasian	Caucasian
Marital status	Single	Single	Single	Divorced	Single	Single
Number of children	None	None	None	2	None	None
Living away from home during term-time?	Yes	Yes	Yes	No	Yes	Yes

**Table 3:** Additional examples of the quotations to illustrate key themes and sub themes.

Theme and Sub-Theme	Examples of Quotations with Code Number
**Studying Dietetics** *Weight*	*‘Obviously I live with dietitians and I’m on a dietetics course so body image is something I’m very aware of’* (B90) *‘The course [has had the biggest influence on diet] obviously because we are dietitians and also more exposure to um all this being healthy thing and because I’ve wanted to lose weight myself and this has been the biggest thing’* (C42) *‘I suppose if I put on weight then I rein it in quite a lot and be quite strict with myself to lose weight again’* (B70) *‘I think the course has made me more conscious of what I eat…I’m quite conscious that I’m quite over weight compared to everyone else on the course…I mean it’s the fact that I’m doing a nutrition based course and even though you’re not supposed to be judgemental, I find I am very judgemental about myself…if you know all these things, you shouldn’t be overweight even though I am* (F40)
**Studying Dietetics** *Knowledge and awareness*	*‘It’s [the dietetics course] made me a lot more conscious and I tend to kind of calorie count now’* (C44) *‘I think the course has made me more conscious of what I eat’* (F34) *‘[I’m] more aware of healthy eating habits so like eating more regularly and things like that. It’s made me think of the evidence base for the supplements and things like that. I think it’s shown me the importance of fruit and vegetables, having your omega 3 and having balanced meals …’* (E48). *‘The course has obviously given us more knowledge in terms of recommendations and being in good health’* (C56)
**Studying Dietetics** *Time*	*‘Probably just not enough time because of the workload and stuff so I’d say probably that’s my number one factor’* (A102) *‘When I lived at home my mum would do the cooking obviously for the evening meals…but I think the extra time for doing that like the extra time going shopping and stuff, i just got lazy to be honest and just stopped, yeah I think and I didn’t put as much effort in as I could really’.* (E62) *‘So cost and time, (pause) time because like I’m really like into my work and I just can’t be bothered sometimes to stand there and cook when I just want to do my work’* (C36) *‘[Lectures] used to run over like lunch so there wasn’t chance to eat then so really that was a bit tough too although I would just end up having to eat in lectures and a lot of the time I would forget to make like um a packed lunch to take in if we were in like around lunch time so then I would just have to buy something from a vending machine that um I could quickly eat and because we didn’t have time to like go to a cafe and get a sandwich or something I would just have to buy like crisps’* (A128) *‘I think it was more healthy before [starting university] because I had the time to prepare it so I was including more meat um probably lunch times were more healthy more salad based, more soup based because I was at home I had the time to prepare fresh soups and salads… also meals get skipped a lot more now just from the point of view of your in university, or you’re getting home too late’* (D52)
**Studying Dietetics** *Stress*	*‘Buying junk food [laugh] and eating it, in times of stress I tend to eat a lot more as well like around exam times when you’re not as active and you are really stressed out you just eat a lot of convenience foods and put a lot of weight on*’ (B78) *‘[Stress] affects me loads, sometimes I’ll eat loads and sometimes I just won’t eat I think it’s the stress of work’* (C156)
**Placement** *Food shopping while at placement*	*‘The main thing that changed was that I couldn’t do the three shops for the food shopping like now’* (F154) *‘When I was in [halls of residence] it wasn’t really a problem because I would finish and walk 5 min to the supermarket and back’* (F148) *‘I um did have my car so it was easy access to the shops so I could buy the weeks shopping and then have structured meals so I would say it had a positive influence in fact’* (A134)
**Placement** *Meal Pattern/Structure while at placement*	*‘...on my first placement I lost quite a lot of weight to be honest just because I wouldn’t plan meals to take with me.’* (E134) *‘Well I found that I didn’t have the time to do as much food preparation’* (F148) *‘I don’t tend to have a cooked meal as much so it’s got worst and with placement and stuff I wasn’t getting home till late so I didn’t want to cook a meal’* (D32) *‘When I used to eat lunch with the dietitians on placement I used to eat more healthily like take a sandwich, salad, fruit or a yoghurt and no chocolate I would have it at home instead’* (F154)
**Influence of significant others** *Peers*	*‘...She would always have a pudding and she would ask if I always wanted to try some...I kinda got into the habit...and this has kinda carried out throughout university.’* (A68) *‘...because she eats a lot she sometimes forces me like saying, ‘is that all you’re eating’ so I feel pressured.’* (C80) *‘Well when I was living with close friends, I had one particular friend who was always on a sort of like a watch of what I was having and she would always be able to tell like if she came into my room and I would hide the things I was having because I wouldn’t want her to see the big bar of chocolate, the sweets and fizzy drinks’* (F118) *‘I did enjoy it, because I did learn more, and I did learn more about flavouring food, whereas before my food before used to be a bit bland like they would come along and be like have you added pepper, have you added salt and I’d be like no, and they would tell me it needs more of this and that so I did learn more about flavouring food. So it’s been positive in that I have learnt to flavour more and cook more meals that I wouldn’t normally have cooked like Indian curry’s and like my previous cooking life I wouldn’t use herbs or spices either and now in my cupboard I’ve got quite a big range.’* (F72)
**Influence of significant others:** *Family*	*‘At home my mom never used to get much rubbish in the house but now I’ve got the freedom and like if everyone else is having them or I just kinda fancy them then I’ll get one so actually it’s kind of a peer thing again I guess (laugh)’* (A104) *‘With the family it’s the case that they chose what I had to eat and when I go home on the weekends now it is like going back to what it was like before university, so I don’t go home very often maybe every 6 weeks or so but now as they are not here by my side they can’t influence me as much.* (F112) *‘Yeah the chocolate I eat now has increased like dramatically [laugh] [pause] Mainly like I said the structure but also because there’s no-one telling me what to eat any more it’s much more my choice’.* (B46) *‘I just had a lot more freedom; it just made me gain weight, a lot of weight’* (F32) *‘Because I have a free rein I can just have whatever I want whenever I want it, it’s a lot more relaxed I can have it when I want it there’s no structure or pattern to my eating it’s just grazing [laugh].* (B30)
**Food Security** *Accessibility*	*‘It relies a lot more on frozen food, probably where I used to do a weekly shop, I don’t usually do the standard big shop on a Saturday every week…partly because of them times when I can’t be bothered to cook, food gets wasted so I think I use the local shops a bit more now, like I’ll think of a recipe and then go to the local shops,* (D86) *‘Yeah it is [food shopping] accessible, because it’s just a walking distance into the town which I’d say is positive and negative, it’s a bit of both I think because you know you can get the food which is a positive but then a negative is if you know you can get it on a daily basis it prevents you from planning what you’d have like you would for a weekly shop’* (B138) *‘I tend to shop at Sainsbury’s now just because I don’t have a car…I just have to go in town but it’s more expensive in Sainsbury’s and I think some of the healthier options are more expensive as well, some of the lower fat options and fruit and veg are really expensive, so I’d say it definitely affects what I buy’* (B120)
**Food Security** *Affordability*	*In my first year, I lived in…self catering and I remember in my first year I used to live on pasta and chicken and vegetables and by the time I had piled it all together there was like a huge mound and I don’t like wasting food and I always made too much, it was quite a lot and I would always end up eating it.’* (F34) *‘Just cost [is the biggest factor] with being on a limited budget now’.* (D30) *‘It [cost] definitely has an impact because say sometimes if you see a muffin that’s like only 50p and a fruit salad that’s like £4 then generally I’d go for the muffin because of the cost of it, so I’d like them to be cheaper places really’.* (A170)
**Food Security** *Availability of Storage space*	*‘I bought a lot of dry food and tins to put in my cupboard rather than fresh food and frozen foods because I just didn’t have room, there were 6 of us and just one small fridge and one small freezer’*. (F54) *‘Well I was making my own food, and what I remember I was making a lot of pasta but then a lot of ready meals as well, but we didn’t have much space in the fridge or freezer so I had to keep going shopping quite a lot and I found that I wasted food quite a bit as well’*. (F52)
**Social, lifestyle and cultural aspects of university life** *Freedom to make food choice*	*[The university experience has had a] ‘Negative, partly because I can eat whatever I want now, but only to realise that actually you can’t you need to keep it in check, like you can enjoy what you like but it can’t be a daily thing like with me it is.’* (F46) *‘I’ve got more freedom, like she [mum] wouldn’t really pick out the unhealthy options where I occasionally do now like I buy biscuits and stuff more where at home we never used to even have them in the house’*. (A82) *‘I thought to myself after 2 weeks I can eat whatever I fancy and no-one has to know, my parents at home wouldn’t be able to go you shouldn’t be eating that, you have to eat this so that’s when it started really.’* (F30)
**Social, lifestyle and cultural aspects of university life** *Eating out, Fast foods, and On campus food establishments*	*‘There’s more take-always near by…and generally I’d think oh I’ve eaten healthily all day so I might as well go and have a take away’* (A34) *‘...I think just outside of university you can get better quality for cheaper cost so I think they are quite expensive especially for students.’* (E78) *‘I find there’s a lot of all you can eat restaurants which is a big factor so because obviously if you’ve been out the night before sometimes you just wanna eat loads and because there are so many around here at uni then you can just go there, pay a fixed amount and can eat as much as you want and generally its quite unhealthy food as well’ (laugh)* (A40) *‘It puts me off buying things from there [on campus food establishments] very often because they are so expensive, if they were cheaper I would definitely use them a bit more’* (F82) *‘I think they [the university food establishments] should make the food cheaper and provide more healthier options in the student cafe’s and places like that like when they say fruit salad’s at these places are either empty they’re past their sell by date’* (F164)
**Social, lifestyle and cultural aspects of university life** *Alcohol*	*‘I kinda drink more often (laugh) like go out more, and cos the drinks are so cheap we drink more on a night out’* (A42) *‘Since I have been at university, I drink a lot, heck of a lot on a night out’* (F102).
**Social, lifestyle and cultural aspects of university life** *Religion and Culture*	*[Alcohol] ‘Its picking up [laugh] becoming a bit better [laugh] and I realise what I’m kinda doing to myself so yeah’* (A38) *‘In first year living with a lot of other people who are complete strangers, they didn’t have the best sort of kitchen hygiene, so it makes me less worried about things like food poisoning being vegetarian’* (B160).
**Social, lifestyle and cultural aspects of university life** *Physical activity*	*‘If I am going running or something then I might have a really late dinner or a really late lunch or I might skip lunch’* (B34) *‘I don’t tend to eat for about 3 or 4 h before I go running and if I am running mid-afternoon then I can’t have lunch and if I am running in the evening then I will have to have a really late dinner. Erm and I get really hungry afterwards so I tend to snack a lot more.’* (B40)
**The future beyond university** Increased awareness of barriers to a healthy diet	*‘I think I’ve got more appreciation of when people turn around and say time as a reason for not doing something, it’s given me more impact on people saying oh I haven’t got enough time, but I think if I was sat the other side of the table talking to me, it would just be planning and organisation, I realise how chaotic it can be working full time and may or may not a single parent to continue to have healthy diets and lifestyles so I think it’s just about being open minded with other people.’* (D152) *‘If they were a uni student and asked me for advice then yeah I would tell them what I think and I would provide ways to help them because I have been through it, I know, I’ve got the experience and I can advise them from what I know and make them aware of little things like on the best places to shop for things’* (C200)

## Data Availability

Not applicable.
